# Editorial: Interdisciplinary approaches to improve quality of soft fruit berries, volume II

**DOI:** 10.3389/fpls.2023.1341519

**Published:** 2023-12-07

**Authors:** Brian Farneti, Lara Giongo, Francesco Emanuelli, Peter Toivonen, Kevin Folta, Massimo Iorizzo

**Affiliations:** ^1^ Berries Genetics and Breeding Unit, Research and Innovation Centre of Fondazione Edmund Mach, San Michele all’Adige (TN), Italy; ^2^ Berrytech srl, Baselga di Pinè (TN), Italy; ^3^ Agriculture and Agri-Food Canada, Summerland Research and Development Centre, Summerland, BC, Canada; ^4^ Horticultural Sciences Department, University of Florida, Gainesville, FL, United States; ^5^ Department of Horticultural Science, Plants for Human Health Institute, North Carolina State University, Kannapolis, NC, United States

**Keywords:** quality, breeding, omics analyses, Vaccinium spp., Rubus spp., postharvest, nutraceutical

Soft fruit berries have gained recognition not only for their delightful taste but also for their potential health benefits. However, to ensure that these benefits reach consumers, it is essential to enhance the fruit quality along the entire production chain (Di Vittori et al., 2018). The quality of soft fruit berries involves a multifaceted array of traits, including visual appeal, overall taste and texture, as well as nutritional attributes. Among these traits, texture, flavor, and appearance exert a direct influence on the fruit’s performance after harvest, its appeal to consumers, and, consequently, its marketability. Delivering high-quality soft fruit berries to consumers requires a comprehensive understanding of their biology, genetics, physiology, and postharvest handling (Klee and Tieman, 2018).

However, the enhancement of fruit flavor and nutritional content has often been left unprioritized in many breeding programs since it was not typically considered a discriminating trait in the early stages of selection (Klee and Tieman, 2018). This new challenge for the breeders is exacerbated by the extensive and time-consuming phenotyping processes commonly used, which makes the analytical screening of large populations of plant material inefficient to support large scale selection.

Within the articles in this Research Topic, the authors deliberate on the importance of developing new tools to support the process of breeding selection. In addition, these studies emphasize innovative technologies and interdisciplinary strategies for quality management across the entire production chain, spanning from the initial breeding selection to the ultimate consumer experience. In particular, authors have discussed the importance of developing chemical and molecular markers to assist breeding selection for three main features: controlling of the phenological stages (Nagasaka et al.), improving quality and storability (Farneti et al., Chizk et al. and Chizk et al.), and enhancing the fruit nutraceutical content (Lafferty et al. and Nguyen et al).

Nagasaka et al. explores the genetic basis of phenology-related traits in southern highbush blueberry (SHB). These berries, adapted to warmer climates, are the result of hybridization between northern highbush blueberries (NHB) and low-chill Vaccinium species. The research employs a genome-wide association study (GWAS) to identify genetic loci associated with phenological traits such as chilling requirement, flowering date, ripening date, fruit development period, and continuous flowering. Results of this study reveal a complex genetic landscape that has shaped the diverse phenological traits in blueberries. Notably, this research identifies the potential introgression of low-chill and late-flowering alleles into the highbush genetic pool. This interdisciplinary approach combines genomics, genetics, and horticulture to provide insights into the genetic basis of blueberry phenology.

The investigation of Farneti et al. sheds light on the role of ethylene in regulating the ripening and storability of blueberries. Understanding ethylene’s impact on soft fruit berries is essential for optimizing preharvest and postharvest strategies to extend shelf life and maintain fruit quality. The research indicates that the production of ethylene varies among different genotypes, suggesting a genotype-specific influence on fruit storage performance, especially on fruit texture. These results support the possibility of tailoring *ad hoc* preharvest and postharvest strategies to extend blueberry shelf life and quality according with the endogenous ethylene production level of each cultivar. In addition, future breeding programs focused on prolonged fruit post-harvest storage need to also consider ethylene production. This can be achieved only with reliable molecular markers and high throughput phenotyping techniques, such as the SRI-ToF-MS methodologies presented in this study.

The study by Chizk et al. addresses the challenge to improve the postharvest quality of blackberries, a soft fruit that often faces challenges during transportation and storage. The research employs genome-wide association studies (GWAS) to identify genetic factors influencing fruit firmness and the occurrence of red drupelet reversion (RDR). Results allowed the identification of several SNPs significantly associated with RDR and fruit firmness. This work demonstrates the complex nature of postharvest quality traits in blackberry, which are likely controlled by many small-effect QTLs. According to the authors’ opinion, given the modest heritability of fruit firmness and RDR, the genomic selection is the most promising long-term strategy for improvement of these traits. Significant QTLs should be used as fixed covariates in a genomic selection model that utilizes existing datasets for model training.

Chizk et al. presents also an innovative software tool called *ShinyFruit*, designed for image-based phenotyping of fruit size, shape, and color-related qualities of blackberry. This tool offers a user-friendly approach to efficiently collect phenotypic data, which is crucial for modern breeding programs. In the context of blackberry breeding, *ShinyFruit* is compared with traditional manual measurements and ImageJ. *ShinyFruit*’s performance in accurately estimating fruit size and color-related qualities demonstrates its potential to enhance phenotyping in fruit breeding programs.

The study of Lafferty et al. delves into the molecular mechanisms behind the coloration of Vaccinium berries, focusing on anthocyanin biosynthesis in bilberry and blueberry. These compounds not only provide color but also contribute to the potential health benefits of these fruits. Transcriptomic analysis in fruit skin and flesh samples across blueberry and bilberry development identified a number of transcription factors strongly correlated with anthocyanin pigmentation in ripening blueberry skin and bilberry skin and flesh. The study identifies the role of MYB transcription factors (TF) as key regulators of anthocyanin production in different berry species. The research emphasizes the coordinated action of MYB activators and repressors, highlighting the complexity of this biological process. In particular, stable overexpression of VcMYBA1 in enhanced the anthocyanin content in transgenic plants, indicating that MYBA1 is sufficient to upregulate the TF module and activate the pathway.

Nguyen’s et al study explores the specific genes involved in anthocyanin production in Vaccinium berries. Anthocyanins are well-known for their health benefits and are responsible for the vibrant colors of these fruits. By isolating and characterizing key genes, the research provides insights into the modulation of anthocyanin production. This interdisciplinary approach combines genetics, biochemistry, and functional genomics to uncover the genes responsible for anthocyanin diversity in soft fruit berries.

These studies collectively underscore the interdisciplinary nature of improving the quality of soft fruit berries. A multi-faceted approach is crucial to optimize the production, flavor, and health benefits of these delicious and nutritious fruits. The findings of these studies not only enhance our understanding of soft fruit biology but also offer valuable tools for breeders to develop superior fruit varieties with extended shelf life and improved quality. In a world where dietary choices increasingly prioritize health and taste, these interdisciplinary efforts promise to deliver better soft fruit berries to our tables. In addition, comprehending how each quality trait remains consistent under various storage and agricultural conditions holds the potential to refine future breeding strategies. These strategies aim to identify accessions that can enhance performance in specific market sectors. Achieving this goal necessitates in-depth research and a close collaboration of analytical methods from diverse fields of expertise.

## Author contributions

BF: Writing – original draft. LG: Writing – review & editing. FE: Writing – review & editing. PT: Writing – review & editing. KF: Writing – review & editing. MI: Writing – review & editing.
